# Parental socioeconomic position and midlife allostatic load: a study of potential mediators

**DOI:** 10.1186/s12889-018-5956-x

**Published:** 2018-08-20

**Authors:** Dinne S. Christensen, Trine Flensborg-Madsen, Ellen Garde, Åse M. Hansen, Jolene M. Pedersen, Erik L. Mortensen

**Affiliations:** 1 Section of Environmental Health, Department of Public Health, University of Copenhagen, Copenhagen, Øster Farimagsgade 5A, 1353 Copenhagen K, Denmark; 2 Center for Healthy Aging, University of Copenhagen, 3B, Blegdamsvej, Copenhagen N,, Denmark; 3 Danish Research Centre for Magnetic Resonance, Centre for Functional and Diagnostic Imaging and Research, Copenhagen University Hospital Hvidovre, Kettegard Allé 30, 2650 Hvidovre, Denmark; 4 Section of Social Medicine, Department of Public Health, University of Copenhagen, Øster Farimagsgade 5, 1014 Copenhagen K, Denmark; 5 National Research Centre for the Working Environment, Lersø Parkallé 105, 2100 Copenhagen, Denmark

**Keywords:** Allostatic load, Parental socioeconomic position, Personality, Social relations, Intelligence, Education

## Abstract

**Background:**

The mechanisms underlying the association of parental socioeconomic position with later life allostatic load remain unclear. The present study aims to examine potential pathways underlying this association: personality, social relations, intelligence and education.

**Methods:**

The study comprised 361 members of the Copenhagen Perinatal Cohort who participated in two subsequent follow-ups: the Prenatal Development Project (mean age 27 years) and the Copenhagen Aging and Midlife Biobank study (mean age 50 years). Allostatic load was based on 14 biomarkers representing the inflammatory, metabolic and cardiovascular system measured at midlife. Information on potential mediators was collected in young adulthood, and their role in the association of parental socioeconomic position with midlife allostatic load were examined in linear regression path analyses.

**Results:**

Parental socioeconomic position at one year was inversely associated with midlife allostatic load (*β* = − 0.238, *p* < .001). No mediation effects were found for personality or social relations. In a model including intelligence and education, a significant indirect effect was found for education (*β* = − 0.151, *p* < .001). A significant direct effect remained (*β* = − 0.111, *p* = .040).

**Conclusions:**

Parental socioeconomic position was inversely associated with allostatic load in midlife. Results suggest that part of this association was mediated by education. A better understanding of the non-cognitive pathways related to education is an important prerequisite for the development of effective intervention strategies.

**Electronic supplementary material:**

The online version of this article (10.1186/s12889-018-5956-x) contains supplementary material, which is available to authorized users.

## Background

Parental or childhood socioeconomic position (SEP) has consistently been found to be associated with later life health and mortality [1–3]. Recently, increasing attention has been paid to the biological mechanisms underlying this association. The concept of allostasis has emerged as an important perspective in studies of how life course exposures to stressful circumstances become biologically embedded. Allostasis refers to the regulatory processes occurring across multiple physiological systems in an (attempted) adaptation to environmental demands. To the extent that these processes become maladaptive over time, they result in so-called allostatic load (AL), reflecting a level of physiological dysregulation associated with disease and premature mortality [4]. Constructed as a multi-system composite index, AL has been shown to predict morbidity and all-cause mortality better than the individual component biomarkers [5], making it useful as an early marker of disease risk [6].

The association of childhood parental SEP with later AL is already well-established [7–10]. The mechanisms underlying this association have been examined to a lesser extent, and identifying potentially modifiable pathways is crucial to the development of effective intervention strategies. Based on theoretical considerations and previous empirical findings, the present study aimed to examine four potential pathways through which parental SEP could influence later AL: personality, social relations, intelligence and education.

The association of personality with AL has received very little attention. However, as AL is posited to reflect physiological wear and tear resulting from chronic stress, individual differences associated with stress exposure, perception and coping are central to the AL model [11]. It is well-known that people with higher levels of neuroticism tend to report more stressors and display more emotional and physiological reactivity to stressors under experimental conditions [12–14]. For this reason, there is a growing interest in the association of personality with AL and inflammation in particular. Cross-sectional studies have shown AL to be positively associated with neuroticism, and inversely with extraversion and conscientiousness [15], while inflammatory markers such as C-reactive protein and interleukin 6, frequently included in the AL construct, have been shown to be inversely associated with conscientiousness and positively with neuroticism [16, 17]. Personality is also associated with parental SEP; low parental SEP is associated with multiple physical and psychosocial risk factors, such as instability, abuse and interpersonal conflict [18, 19], and children growing up in low SEP environments have been found to have more psychosocial difficulties and higher risk of psychiatric illness [20, 21]. Parental SEP is thus likely to affect non-morbid personality development as well, and a few studies have shown associations of parental SEP with adult personality scores [22–25].

Personality is also associated with social relations, and several studies have shown various aspects of social relations to be associated with both SEP and AL. Low parental education and adult SEP have been found to be associated with lower levels of social support [26, 27]. Social support, frequency of social contact and other measures of social relations, such as self-reported negative experiences with spouse and family, have also been found to be associated with AL after adjusting for age, education and health behaviors [28, 29].

The adverse exposures associated with growing up in a low SEP environment have also been found to have a detrimental effect on neurocognitive development [30, 31], and parental SEP has consistently been found to be positively associated with intelligence and educational achievement [32–36], perhaps because high SEP parents provide more cognitively stimulating environments, e.g. by reading more frequently to their children [37]. Education and intelligence can be hypothesized to affect AL in several ways. First, some studies have shown higher education and intelligence to be associated with lower exposure to stressful and traumatic events [38–40]. Second, cognitive functions are related to stress perception and coping. For example, measures of working memory have been found to be positively associated with successful emotion regulation and neutral appraisals of a negative emotional stimulus [41, 42], and general daily stressors have been associated with higher negative affect in people with mild cognitive impairment compared to controls [43]. Such findings support an understanding of stress exposure, perception and management as processes which to some extent depend on cognitive and non-cognitive stable characteristics of the individual. Both cognitive ability and education have also been shown to be associated with later AL. In the Lothian Birth Cohort, a broad measure of intelligence was inversely associated with AL more than 50 years later [44, 45], and Gale et al. [46] found choice reaction time at age 16 to be positively associated with AL at age 36. Related to intelligence, education has been shown to influence health not only through related gains in occupational achievement and wealth, but also by increasing health literacy and promoting health behavior [47]. One of the earliest studies of AL showed an inverse association of educational attainment with AL [48], and recent findings have suggested that education mediates the association of childhood SEP with adult AL [49, 50].

Barboza Solís et al. [50] recently examined several potential mediators of the association of parental SEP with AL at age 44 and found educational attainment at age 23 to be a primary mediator. However, as the authors noted, the study was limited by lack of information on variables representing potential pathways, such as cognitive ability and personality traits. Using prospectively measured data from infancy, young adulthood and midlife in formal path analyses, the present study aimed to test these and other mediators of the parental SEP-AL association. We hypothesized an inverse association between parental SEP and AL, partly mediated by personality traits, satisfaction with social relations, intelligence level and years of education.

## Methods

### Study sample

The study sample consists of 361 members of the Copenhagen Perinatal Cohort (CPC, 1959–1961) who participated in two subsequent follow-up studies: the Prenatal Development Project (PDP, 1982–1994) at the mean age of 27 years (SD = 4.36), and the Copenhagen Aging and Midlife Biobank study (CAMB, 2009–2011) at the mean age of 50 years (SD = 0.79).

The CPC consists of 9125 children born to 8949 mothers at the Copenhagen University Hospital between October 1959 and December 1961. Admittance was based on area of residence (Copenhagen), but some were referred due to obstetrical complications or single mother status [51]. Information on pre-, peri- and postnatal factors was collected in personal interviews during pregnancy, at birth and at a 1-year follow-up assessment.

The PDP focused on the long-term effects of pre- and perinatal factors on psychological and physical development [52]. On the basis of perinatal records, 1575 members of the CPC were invited to the PDP which included personality assessment by the Eysenck Personality Questionnaire (EPQ) [53], intelligence assessment by the Danish version of the Wechsler Adult Intelligence Scale (WAIS) [54], and extensive questionnaires on physical, psychological and social factors. A total of 1208 members of the CPC agreed to participate in the PDP.

In 2009, 843 of the PDP participants were invited to the CAMB study [55] including questionnaires and a health examination with blood samples. The final sample consisted of 361 members of the CPC with continued participation in the PDP and information on AL from CAMB (Additional file 1: Figure S1: Overview of the data collection).

The CAMB study protocol was approved by the local ethics committee (No: H-A-2008-126) and Danish Data Protection Agency (No: 2008–41-2938) and all participants signed informed consent forms.

### Measures

#### Allostatic load

AL was measured as an index score based on 14 biomarkers extracted from non-fasting blood samples and representing three regulatory systems: The inflammatory system (interleukin 6, tumor necrosis factor α, high sensitivity C-reactive protein), the metabolic system (low density lipoprotein, high density lipoprotein, total cholesterol, body mass index (BMI), waist/hip ratio, blood glucose, triglycerides, HbA1c, percent body fat) and the cardiovascular system (systolic and diastolic blood pressure, averaged across four measurements). A detailed description of data collection and blood sample analyses can be found in Hansen et al. [56]. The AL score was computed using the traditional count-based method of summing the number of AL markers falling in the high-risk quartile [4], resulting in an index range of 0–14. Cut-offs were derived from the full CPC subsample of CAMB (*N* = 1718) and defined as the within-sample sex-specific 75th percentile, except in the case of HDL, for which high risk was defined as below the 25th percentile (Additional file 2: Table S1: Sex-stratified means, medians and risk cut-points for AL biomarkers). Information on all 14 biomarkers was available for 97% of the CPC subsample participants. AL scores were computed only for participants with information on at least 50% of the included biomarkers, resulting in the exclusion of seven participants with ≥8 biomarkers missing. For participants with at least seven but less than all 14 biomarkers available, AL was computed by multiplying the mean score of the available biomarkers by 14.

#### Parental socioeconomic position

Parental SEP was based on information from the 1-year follow-up examination and was derived from information on four factors, each of which were assigned points from 0 to 5: occupation of the breadwinner, type of income of the breadwinner (e.g. unemployment relief, weekly or monthly wage, own business or capital etc.), education of the breadwinner and quality of living accommodation (size, number of persons per room etc.). In relation to the data collection, the resulting 0–20 point scale was recoded to a scale from 1 to 8, with lower values reflecting lower levels of SEP [51].

#### Young adulthood mediators

Potential mediators from the PDP follow up (mean age 26.9 years) were selected a priori based on theoretical considerations and previous findings.

##### Personality

Personality traits were measured using a Danish version of the Eysenck Personality Questionnaire (EPQ) [53]. The questionnaire consisted of 101 ‘yes’ or ‘no’ response items. From these, scores on trait neuroticism, extraversion, psychoticism and social desirability (lie scale [57, 58]) were derived.

##### Intelligence

Intelligence was measured using the complete Wechsler Adult Intelligence Scale (WAIS) [54]. The test was individually administered by three psychologists, all of whom were blinded to information on parental SEP and other early life factors of the participants. Danish test score norms were used to derive Full Scale IQ scores. Sample range was 64–138.

##### Years of education

The highest level of primary and secondary school education of the participants was recorded in personal interviews and coded as years of education. Sample range was 7–13 years.

##### Social relations satisfaction

Two scales were created to reflect the participants’ satisfaction with their social relations. Social relations satisfaction: In personal interviews, participants were asked: “Can you mention some aspects of life that you are particularly satisfied with?”. Based on the answers, a list of items representing different aspects of life was produced, including three items about social relations with family, partner and friends, respectively. For each of these items, a binary variable was created indicating whether the item was mentioned. A social relations satisfaction score was constructed as the sum of these binary variables with a sample score range of 0–3. Social relations dissatisfaction: Participants were asked: “Can you mention some aspects of life that you are particularly dissatisfied with?”. Again, a binary variable was created for each item; family, partner and friends, indicating whether or not this was mentioned as an area of particular dissatisfaction. The range for the dissatisfaction score was 0–2.

#### Covariates

In all mediation models, potential mediators were adjusted for sex and age at young adulthood follow-up (range 20–34 years). Additionally, AL was adjusted for time of day of blood draw and fasting status within two hours of blood draw to account for natural diurnal variation in some biomarkers. For the purpose of supplementary analyses, potential early life biomedical confounders were selected among factors found to be associated with childhood SEP and recently found to predict midlife AL in the Perinatal Cohort (unpublished observations). These were: maternal smoking in the third trimester (yes/no), complications at birth (yes/no) and maternal BMI. Further, the effect of adult SEP measured concurrently with the AL biomarkers was examined in all mediation models. Adult SEP was based on occupational social class coded I to VI with I representing professional occupation and VI representing transfer income [59].

### Statistical analyses

First, the association of parental SEP with AL was assessed in a model containing no mediators or confounders. Next, the effects of potential mediators on the parental SEP-AL association were tested in three separate linear regression path analysis models: Model 1: extraversion, neuroticism, psychoticism and lie scale, Model 2: social relations satisfaction and dissatisfaction, and Model 3: intelligence and school education. Finally, in Model 4, all potential mediators were combined in a single model. In supplementary analyses, all models were adjusted for adult SEP and potential confounding factors related to parental SEP and known to influence adult AL. All variables were standardized before analyses.

Sex differences were examined using independent samples *t*-tests. Potential interaction effects of sex on the parental SEP-AL association were examined in multiple regression analyses and revealed no significant interaction terms. Missing data rate ranged from 0% (AL, sex) to 10.8% (social relations satisfaction and dissatisfaction scores). To handle missing data, the full information maximum likelihood (FIML) procedure was used, enabling use of all available information [60]. All analyses were also conducted using only complete cases. Robust standard errors were used in all mediation models, and all analyses were performed in Stata V14.

## Results

Table 1 shows means and standard deviations for AL and potential mediators and covariates, stratified by low and high parental SEP, and *p*-values for tests of differences according to parental SEP group. The sample comprised 53.5% women. Participants with low parental SEP at one year had higher levels of AL at midlife, fewer years of education and lower levels of intelligence (*p*-values for *t*-tests < .001).

**Table 1:** Descriptive statistics of parental SEP, allostatic load, potential mediators and covariates including tests of differences according to parental SEP group

	Low parental SEP (1–5)		High parental SEP (6–8)		Full sample	
	*n*	Mean (SD)	*n*	Mean (SD)	*n*	*p* ^a^
Allostatic load (midlife)	208	4.05 (2.73)	123	2.72 (2.33)	361	< .001
Parental SEP (infancy)	208	3.56 (1.04)	123	6.85 (0.77)	331	< .001
Potential mediators (young adulthood)						
EPQ neuroticism [0–23]	185	7.58 (5.28)	116	6.91 (4.61)	326	.26
EPQ extraversion [0–21]	185	14.7 (4.30)	116	15.1 (4.07)	327	.38
EPQ psychoticism [0–25]	184	4.07 (2.41)	116	4.20 (2.19)	326	.65
EPQ lie scale [0–21]	186	7.30 (3.24)	115	6.70 (3.42)	327	.13
Social relations satisfaction	182	0.75 (0.80)	114	0.80 (0.84)	322	.64
Social relations dissatisfaction	182	0.10 (0.32)	114	0.13 (0.36)	322	.50
Intelligence	184	101.5 (13.7)	115	110.5 (13.5)	325	< .001
Years of education	184	10.6 (1.48)	115	11.9 (1.48)	325	< .001
Covariates						
Female sex (%)	113	54.3	67	54.5	361	.98
Young adulthood age	184	26.9 (4.23)	115	26.6 (4.40)	325	.54
Time of blood draw	207	11.2 (2.30)	123	11 (2.37)	360	.29
Fasting (%)	139	66.8	86	69.9	359	.49
Maternal smoking (%)	87	41.8	41	33.3	354	.14
Complications at birth (%)	16	7.69	10	8.13	360	.90
Maternal BMI	189	22.2 (3.36)	117	21.3 (2.26)	334	.014
Adult SEP	204	3.24 (1.45)	118	2.49 (1.39)	352	< .001

*Note*. SEP = Socioeconomic position. *EPQ* = Eysenck Personality Questionnaire. *BMI* = Body Mass Index

^a^*t*-tests or chi-square tests

In accordance with previous studies, there was a highly significant inverse bivariate association of parental SEP with AL (*r* = −.26, *p* < .001) (Additional file 3: Table S2: Correlation matrix of all study variables). Also significantly associated with parental SEP were intelligence and years of education (*p* < .001). AL was significantly inversely associated with intelligence and years of education, and marginally significantly associated with extraversion (*p* = .074) and social relations satisfaction (*p* = .079).

In a model adjusted only for fasting status and time of blood draw, the association of parental SEP with midlife AL was *β* = − 0.238, *p* < .001. The model R-squared showed that this model explained 9.2% of the variance in AL.

### Model 1: Mediational effects of neuroticism, extraversion, psychoticism, and lie scale

**Fig. 1:**
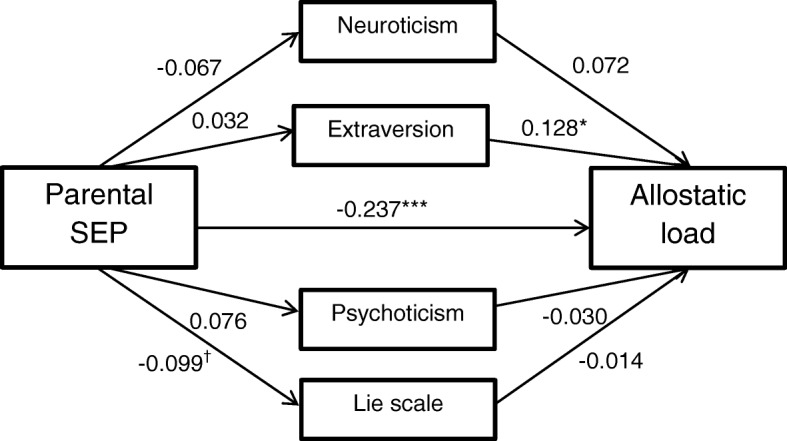
Direct and indirect paths in Model 1. Mediators adjusted for sex and young adulthood age. AL adjusted for fasting status within two hours before blood draw and time of blood draw. Path coefficients are standardized. ^†^*p* < .10; *p < .05; **p < .01; ***p < .001

Figure 1 shows a path diagram of Model 1. Contrary to our hypothesis, none of the examined personality traits significantly mediated the parental SEP-AL association. Aside from the association of extraversion with AL (*β* = 0.128, *p* = .024) and the direct effect of parental SEP on midlife AL (*β* = − 0.237, *p* < .001), there were no significant paths in this model. Model 1 explained approximately 11% of the variance in AL.

### Model 2: Mediational effects of social relations satisfaction and dissatisfaction

**Fig. 2:**
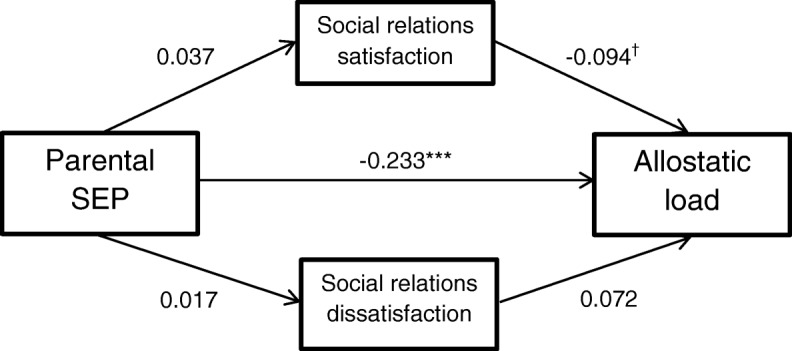
Direct and indirect paths in Model 2. Mediators adjusted for sex and young adulthood age. AL adjusted for fasting status within two hours before blood draw and time of blood draw. Path coefficients are standardized. ^†^*p* < .10; * *p* < .05; **p < .01; ***p < .001

Figure 2 shows a path diagram of Model 2. While the direct effect of social relations satisfaction on AL was marginally significant (*β* = − 0.094, *p* = .066), neither social relations satisfaction nor dissatisfaction significantly mediated the parental SEP-AL association. The model explained 11% of the variance in AL.

### Model 3: Mediational effects of intelligence and years of education

**Fig. 3:**
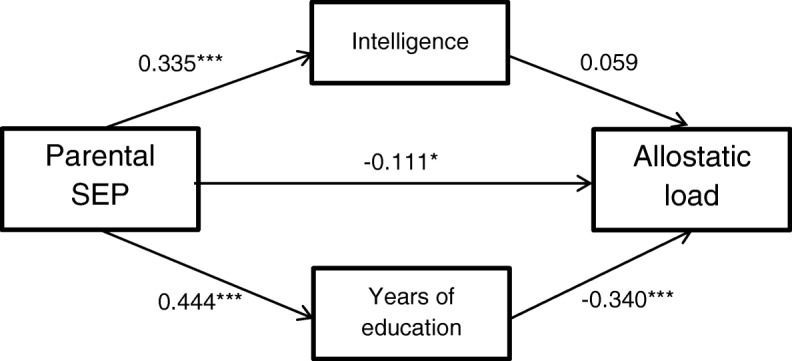
Direct and indirect paths in Model 3. Mediators adjusted for sex and young adulthood age. AL adjusted for fasting status within two hours before blood draw and time of blood draw. Path coefficients are standardized. ^†^*p* < .10; *p < .05; **p < .01; ***p < .001

Figure 3 shows a path diagram of Model 3. In this model, the total effect of parental SEP on AL (*β* = − 0.242, *p* < .001) was significantly mediated by years of education (*β* = − 0.151, *p* < .001). Despite a highly significant path from parental SEP to intelligence, the path from intelligence to AL was not statistically significant (*β* = 0.059, *p* = .33). A direct effect of parental SEP on AL remained (*β* = − 0.111, *p* = .040). Model 3 explained 17% of the variance in AL.

### Model 4: Full model containing all potential mediators

**Fig. 4:**
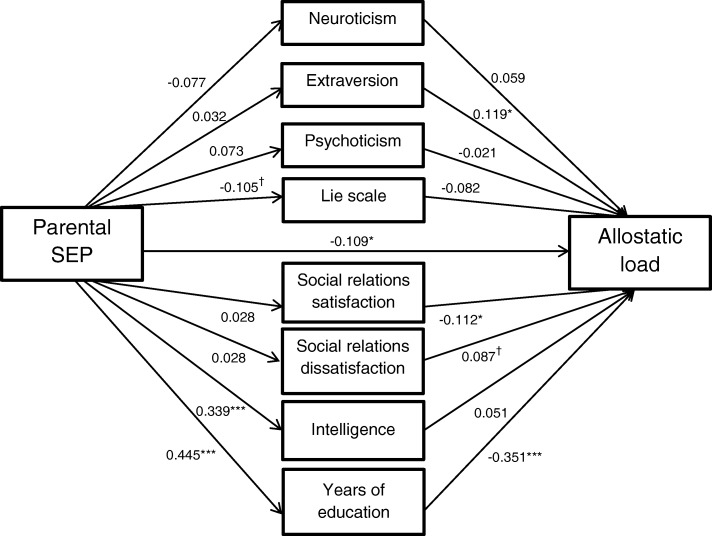
Direct and indirect paths in Model 4. Mediators adjusted for sex and young adulthood age. AL adjusted for fasting status within two hours before blood draw and time of blood draw. Path coefficients are standardized. ^†^*p* < .10; *p < .05; **p < .01; ***p < .001

Figure 4 shows a path diagram of Model 4 with all potential mediators in a combined model. The associations of social relations satisfaction and dissatisfaction with AL were strengthened (*β* = − 0.112, *p* = .019 and *β* = 0.087, *p =* .078, respectively), but their indirect effects remained non-significant. This model explained 22% of the variance in AL.

Table 2 shows a partitioning of the direct and indirect effects for all four models.

**Table 2:** Indirect and direct effects of parental SEP on midlife allostatic load, adjusted for covariates (*N* = 361)

	*β* [CI]ª	*p*
Model 1		
Indirect effects	− 0.002 [− 0.023, 0.019]	.87
EPQ neuroticism	− 0.005 [− 0.015, 0.006]	.36
EPQ extraversion	0.004 [− 0.011, 0.019]	.59
EPQ psychoticism	−0.002 [− 0.011, 0.006]	.61
EPQ lie scale	0.001 [− 0.009, 0.012]	.80
Direct effect	− 0.237 [− 0.339, − 0.136]	< .001
Total effect	−0.239 [− 0.340, − 0.138]	< .001
Model 2		
Indirect effects	−0.002 [− 0.019, 0.014]	.79
Social relations satisfaction	−0.003 [− 0.015, 0.008]	.53
Social relations dissatisfaction	0.001 [−0.009, 0.011]	.81
Direct effect	−0.233 [− 0.333, − 0.132]	< .001
Total effect	− 0.235 [− 0.337, − 0.133]	< .001
Model 3		
Indirect effects	−0.131 [− 0.187, − 0.075]	< .001
Intelligence	0.020 [− 0.021, 0.061]	.34
Years of education	−0.151 [− 0.214, − 0.088]	< .001
Direct effect	−0.111 [− 0.217, − 0.005]	.040
Total effect	−0.242 [− 0.343, − 0.141]	< .001
Model 4		
Indirect effects	−0.133 [− 0.193, − 0.074]	< .001
EPQ neuroticism	− 0.005 [− 0.014, 0.005]	.37
EPQ extraversion	0.004 [− 0.010, 0.017]	.58
EPQ psychoticism	−0.002 [− 0.009, 0.006]	.70
EPQ lie scale	0.009 [−0.006, 0.023]	.24
Social relations satisfaction	−0.003 [− 0.016, 0.009]	.62
Social relations dissatisfaction	0.002 [−0.009, 0.014]	.68
Intelligence	0.017 [−0.024, 0.058]	.42
Years of education	−0.156 [− 0.218, − 0.094]	< .001
Direct effect	− 0.109 [− 0.210, − 0.007]	.036
Total effect	−0.242 [− 0.343, − 0.141]	< .001

*Note*. SEP = Socioeconomic position. *EPQ* = Eysenck Personality Questionnaire

^a^Standardized beta coefficients reported

Two supplementary analyses were conducted: first, we adjusted AL for midlife SEP measured concurrently with the biomarker collection in the CAMB study. This strengthened the association of social relations satisfaction with AL in Model 2 (*β* = − 0.103, *p* = .046) and slightly attenuated the direct association of parental SEP with AL in all models; in Model 3 (*β* = − 0.100, *p* = .068) and model 4 (*β* = − 0.097, *p* = .064) to marginal significance. The adjustment for adult SEP did not significantly change results regarding mediation. Second, we adjusted for potential confounders of the associations between potential mediators and AL related to early life health. Potential confounders were maternal smoking in the third trimester, complications at birth and maternal BMI. While the overall results were unchanged, this did attenuate some estimates; in particular, in Model 3 the direct association of parental SEP with AL was reduced to marginal significance (*β* = − 0.105, *p* = .052). Aside from this, there were no substantial changes in the results. Using a complete case approach yielded no significant differences in results for any model.

## Discussion

In this study, parental SEP at one year was inversely associated with AL at midlife. Several potential mediators of this association were tested, but significant mediation was found only for years of school education. The direct effect of parental SEP remained in all models.

In Model 1 the role of neuroticism, extraversion, psychoticism and lie scale personality traits were examined, which to our knowledge have not previously been tested as mediators of the parental SEP-AL association. The finding that none of the included personality traits mediated the parental SEP-AL association was surprising considering the central role of stress exposure and experience in AL theory, and the well-established association of personality with stress exposure and coping [12, 61]. It is however consistent with a study examining a range of personality traits as mediators of the adult SEP-AL association, including hostility, agreeableness, emotional stability and surgency [62]. Significant mediation was found only for hostility, which has also been found to mediate the association of education with AL [48]. Thus, the inclusion of more specific facet-measures of personality, such as hostility, or of different traits, such as conscientiousness, might have yielded different results. Further, as most of the direct associations among parental SEP, personality traits and AL were in the expected directions (e.g. lower levels of parental SEP related to higher levels of neuroticism, in turn related to higher levels of AL), we do suspect some of our results to be influenced by our sample size and resulting power issues; a previous study based on a larger sample of the PDP study (*N* = 1055) found significant associations of parental SEP with neuroticism, psychoticism and lie scale [23].

Model 2 showed no indication of a mediating effect of social relations satisfaction or dissatisfaction. This is in accordance with studies of the effects of similar mediators such as social support and strain [63], although some findings do suggest that adult social relationships can offset the effect of childhood socioeconomic and social adversity on AL to some extent [7, 64].

Model 3 showed the effect of parental SEP on midlife AL to be partially mediated by years of education. This confirms previous findings of educational attainment mediating the effect of early life socioeconomic adversity on midlife AL [49, 50, 63]. A recent study examined four different pathways in the associations of maternal education and paternal occupation at birth with AL at age 44 years; an educational, a psychosocial, a financial and a health behaviors pathway. In accordance with our findings, the primary mediator of both measures of parental SEP was educational attainment at age 23 years [50]. The authors hypothesized an underlying effect of a cognitive construct that they were unable to test directly. In the present study, using the WAIS Full Scale IQ score allowed us to test the mediating role of such a construct; while there was a borderline significant indirect effect of intelligence in a model with this as the only mediator, this effect was strongly attenuated in Model 3 when including years of education. Despite highly significant associations of parental SEP with both years of education and intelligence, only years of education was significantly associated with AL in a combined model, suggesting that mediation occurs via non-cognitive factors related to education.

This is, however, in contrast to results from several studies of AL reporting a minor role of education compared to intelligence. In the Dunedin cohort, childhood IQ measures collected before the onset of formal schooling significantly predicted midlife scores on a biomarker algorithm similar to AL [65]. While the effect of later education was not estimated, this was examined in the Lothian Birth Cohort [45]; adjusting for age 11 IQ, parental SEP was no longer a significant predictor of AL at age 70, and the association of IQ with AL was only partially mediated by educational attainment. These findings suggest that early measures of intelligence significantly (and independently of education) predict AL. Aside from the smaller sample size of the present study, the discrepancy with the present findings could result from cohort effects, reflecting generational differences in the effect of education on AL. In a recent twin study [66], the within-pair effect of different educational levels on AL was found to be non-significant in monozygotic twins. While the sample age range (37–86 years) was considerably broader than that of the present study, again limiting comparability, the finding highlights the possibility of genetic or environmental confounding. The present study tested potential confounding of the education-AL association by maternal smoking, complications at birth and maternal BMI, none of which significantly attenuated the mediating effect of education.

The question thus remains which non-cognitive factors related to education might influence AL. Potential candidates can be found in factors associated with education but not included in the present study. First, educational attainment is associated with health-related behavior [67], and returning to the role of personality, education is positively associated with trait conscientiousness and an internal locus of control [68, 69], both associated with better health behaviors [70]. Education is also inversely associated with hostility, which has been shown to mediate the association of education with AL [48]. Second, education is inversely associated with perceived social status, which has been found to be associated with AL [71]. Thus, it is possible that part of the effect of education on AL arises through the perception of social inequality. Finally, education may influence AL through lower stress exposure, as it is positively associated with both socioeconomic success and conscientiousness [27, 72, 73]. However, evidence is limited for mediation of the of parental SEP-adult AL association by health behaviors and adult stress exposure [7, 9, 45, 50], and findings regarding the role of adult SEP are inconsistent, with some studies showing a stronger effect on AL of childhood SEP measures [10], and others of adulthood SEP [7, 74]. In this study, the direct effect of parental SEP was slightly attenuated after adjusting for adult SEP, while the indirect effect of education on AL persisted. Further research into the pathways linking education and AL seems warranted.

Finally, while the present findings suggest that the effect of parental SEP on AL is partly mediated by educational achievement, a substantial part of the effect remained unexplained in the present and other studies [7, 9, 50]. Whether this reflects an indirect path through behavioral or environmental factors not included or adequately represented in this study, or an independent (perhaps genetically confounded or biologically embedded) effect remains unknown. It is also possible that the effect of parental SEP observed in the present study is in fact an effect of the risks and adverse experiences associated with low SEP, which have previously been found to predict AL [63, 75, 76]. We find it most likely that these explanations are not mutually exclusive, but that longitudinal associations such as those in the present study arise in an interplay of direct and indirect socioeconomic and psychosocial factors [77].

### Strengths and limitations

The primary strengths of the present study are the use of prospective data and the combination of specific pathways not previously examined in the context of parental SEP and AL. The parental SEP measure was based on multiple indicators reflecting both economic and social factors. However, although the factors examined as potential mediators have been selected based on their association with both parental SEP, stress and AL, the lack of more direct measures of stress exposure or adverse experiences is a limitation, considering the conceptualization of AL as reflecting the consequences of chronic stress exposure.

Further, by using a multifaceted preclinical outcome measure in a midlife population, we have sought to minimize survival bias and focus on predisease pathways [6], though this does limit generalizability to older populations. Generalizability is additionally limited by the fact that parental SEP in infancy was higher in the present sample (i.e. among participants in the young adulthood and midlife follow-up) compared to the full Copenhagen Perinatal Cohort (*p* < .001).

While previous findings have highlighted the problem of reverse causation when studying predictors of outcomes related to AL [78–80], this seems unlikely in the present context, given that the included mediators have on average been measured more than 20 years prior to the assessment of AL biomarkers. Finally, our measure of AL did not include any neuroendocrine markers. As the HPA axis activity related to the neuroendocrine system is posited to play a central role in the stress response associated with AL, we run the risk of underestimating some effects which might have been detected using a more comprehensive measure of AL [81].

## Conclusions

Using prospective data from infancy, young adulthood and midlife, the present study examined potential pathways in the well-established association of parental SEP with adult allostatic load. No indirect effects were found for social relations satisfaction or personality, which was not previously examined as a potential mediator of the parental SEP-AL association. Supporting previous findings, a significant indirect effect was found for years of school education. The fact that no such effect was found for intelligence suggests mediation by non-cognitive factors related to education. The findings thus highlight the need for further research into the mechanisms by which education reduces the risk of physiological dysregulation.

## Additional files

Additional file 1: **Figure S1.** Overview of the data collection. (PDF 73 kb)

Additional file 2: **Table S1.** Sex-stratified means, medians and risk cut-points for AL biomarkers. (DOCX 20 kb)

Additional file 3: **Table S2.** Correlation matrix of all study variables. (XLSX 16 kb)

## Abbreviations

AL: Allostatic load
BMI: Body Mass Index
CAMB: Copenhagen Aging and Midlife Biobank
CPC: Copenhagen Perinatal Cohort
EPQ: Eysenck Personality Questionnaire
FIML: Full Information Maximum Likelihood
PDP: Prenatal Development Project
SEP: Socioeconomic position
WAIS: Wechsler Adult Intelligence Scale

## References

[1] Cohen S, Janicki-Deverts D, Chen E, Matthews KA (2010). Childhood socioeconomic status and adult health. Ann N Y Acad Sci.

[2] Galobardes B, Lynch JW, Smith GD (2004). Childhood socioeconomic circumstances and cause-specific mortality in adulthood: systematic review and interpretation. Epidemiol Rev.

[3] Miller GE, Chen E, Parker KJ (2011). Psychological stress in childhood and susceptibility to the chronic diseases of aging: moving towards a model of behavioral and biological mechanisms. Psychol Bull.

[4] Seeman TE, Singer BH, Rowe JW, Horwitz RI, McEwen BS (1997). Price of adaptation—allostatic load and its health consequences: Macarthur studies of successful aging. Arch Intern Med.

[5] Beckie TM (2012). A systematic review of allostatic load, health, and health disparities. Biol Res Nurs.

[6] Singer BH, Ryff CD (2001). Health NRC (US) C on FD for B and SSR at the NI of.

[7] Gruenewald TL, Karlamangla AS, Hu P, Stein-Merkin S, Crandall C, Koretz B (2012). History of socioeconomic disadvantage and allostatic load in later life. Soc Sci Med 1982.

[8] Evans GW, Kim P (2012). Childhood poverty and young adults’ Allostatic load the mediating role of childhood cumulative risk exposure. Psychol Sci.

[9] Turner RJ, Thomas CS, Brown TH (2016). Childhood adversity and adult health: Evaluating intervening mechanisms. Soc Sci Med 1982.

[10] Robertson T, Popham F, Benzeval M (2014). Socioeconomic position across the lifecourse & allostatic load: data from the west of Scotland Twenty-07 cohort study. BMC Public Health.

[11] McEwen BS (2002). Sex, stress and the hippocampus: allostasis, allostatic load and the aging process. Neurobiol Aging.

[12] Magnus K, Diener E, Fujita F, Pavot W (1993). Extraversion and neuroticism as predictors of objective life events: a longitudinal analysis. J Pers Soc Psychol.

[13] Boals A, Southard-Dobbs S, Blumenthal H (2015). Adverse events in emerging adulthood are associated with increases in neuroticism. J Pers.

[14] Bolger N, Schilling EA (1991). Personality and the problems of everyday life: the role of neuroticism in exposure and reactivity to daily stressors. J Pers.

[15] Stephan Y, Sutin AR, Luchetti M, Terracciano A (2016). Allostatic load and personality: a 4-year longitudinal study. Psychosom Med.

[16] Luchetti M, Barkley JM, Stephan Y, Terracciano A, Sutin AR (2014). Five-factor model personality traits and inflammatory markers: new data and a meta-analysis. Psychoneuroendocrinology.

[17] Sutin AR, Terracciano A, Deiana B, Naitza S, Ferrucci L, Uda M (2010). High neuroticism and low conscientiousness are associated with Interleukin-6. Psychol Med.

[18] Evans GW (2004). The environment of childhood poverty. Am Psychol.

[19] Schickedanz A, Dreyer BP, Halfon N (2015). Childhood poverty: understanding and preventing the adverse impacts of a most-prevalent risk to pediatric health and well-being. Pediatr Clin N Am.

[20] Bradley RH, Corwyn RF (2002). Socioeconomic status and child development. Annu Rev Psychol.

[21] Duncan GJ. In: Brooks-Gunn J, editor. Consequences of Growing Up Poor: Russell Sage Foundation; 1997. http://www.jstor.org/stable/10.7758/9781610448260. Accessed 14 Dec 2017.

[22] Carver CS, Johnson SL, McCullough ME, Forster DE, Joormann J. Adulthood personality correlates of childhood adversity. Front Psychol. 2014;5 10.3389/fpsyg.2014.01357. 10.3389/fpsyg.2014.01357 PMC4240049 25484874

[23] Flensborg-Madsen T, Mortensen EL (2014). Infant SES as a predictor of personality—is the association mediated by intelligence?. PLoS One.

[24] Harper S, Lynch J, Hsu W-L, Everson SA, Hillemeier MM, Raghunathan TE (2002). Life course socioeconomic conditions and adult psychosocial functioning. Int J Epidemiol.

[25] Jonassaint CR, Siegler IC, Barefoot JC, Edwards CL, Williams RB (2011). Low life course socioeconomic status (SES) is associated with negative NEO PI-R personality patterns. Int J Behav Med.

[26] Felner RD, Brand S, DuBois DL, Adan AM, Mulhall PF, Evans EG (1995). Socioeconomic disadvantage, proximal environmental experiences, and socioemotional and academic adjustment in early adolescence: investigation of a mediated effects model. Child Dev.

[27] Evans GW, Kim P (2010). Multiple risk exposure as a potential explanatory mechanism for the socioeconomic status–health gradient. Ann N Y Acad Sci.

[28] Brooks KP, Gruenewald T, Karlamangla A, Hu P, Koretz B, Seeman TE (2014). Social relationships and allostatic load in the MIDUS study. Health Psychol Off J Div Health Psychol Am Psychol Assoc..

[29] Seeman TE, Singer BH, Ryff CD, Dienberg Love G, Levy-Storms L (2002). Social relationships, gender, and allostatic load across two age cohorts. Psychosom Med.

[30] Blair C (2010). Stress and the development of self-regulation in context. Child Dev Perspect.

[31] Noble KG, Norman MF, Farah MJ (2005). Neurocognitive correlates of socioeconomic status in kindergarten children. Dev Sci.

[32] Kaplan GA, Turrell G, Lynch JW, Everson SA, Helkala EL, Salonen JT (2001). Childhood socioeconomic position and cognitive function in adulthood. Int J Epidemiol.

[33] Turrell G, Lynch JW, Kaplan GA, Everson SA, Helkala E-L, Kauhanen J (2002). Socioeconomic position across the lifecourse and cognitive function in late middle age. J Gerontol B Psychol Sci Soc Sci.

[34] Hackman DA, Betancourt LM, Gallop R, Romer D, Brodsky NL, Hurt H (2014). Mapping the trajectory of socioeconomic disparity in working memory: parental and neighborhood factors. Child Dev.

[35] Sirin SR (2005). Socioeconomic status and academic achievement: a meta-analytic review of research. Rev Educ Res.

[36] von Stumm S, Plomin R (2015). Socioeconomic status and the growth of intelligence from infancy through adolescence. Intelligence.

[37] Gibbs BG, Forste R (2014). Breastfeeding, parenting, and early cognitive development. J Pediatr.

[38] Hatch SL, Dohrenwend BP (2007). Distribution of traumatic and other stressful life events by race/ethnicity, gender, SES and age: a review of the research. Am J Community Psychol.

[39] Breslau N, Davis GC, Andreski P, Peterson E (1991). Traumatic events and posttraumatic stress disorder in an urban population of young adults. Arch Gen Psychiatry.

[40] Breslau N, Lucia VC, Alvarado GF (2006). Intelligence and other predisposing factors in exposure to trauma and posttraumatic stress disorder: a follow-up study at age 17 years. Arch Gen Psychiatry.

[41] Opitz PC, Lee IA, Gross JJ, Urry HL (2014). Fluid cognitive ability is a resource for successful emotion regulation in older and younger adults. Front Psychol.

[42] Schmeichel BJ, Volokhov RN, Demaree HA (2008). Working memory capacity and the self-regulation of emotional expression and experience. J Pers Soc Psychol.

[43] Rickenbach EH, Condeelis KL, Haley WE (2015). Daily stressors and emotional reactivity in individuals with mild cognitive impairment and cognitively healthy controls. Psychol Aging.

[44] Booth T, Royle NA, Corley J, Gow AJ, Valdés Hernández Mdel C, Muñoz Maniega S (2015). Association of allostatic load with brain structure and cognitive ability in later life. Neurobiol Aging.

[45] Gale CR, Booth T, Starr JM, Deary IJ (2016). Intelligence and socioeconomic position in childhood in relation to frailty and cumulative allostatic load in later life: the Lothian birth cohort 1936. J Epidemiol Community Health.

[46] Gale CR, Batty GD, Cooper S-A, Deary IJ, Der G, BS ME (2015). Reaction time in adolescence, cumulative Allostatic load, and symptoms of anxiety and depression in adulthood: the west of Scotland Twenty-07 study. Psychosom Med.

[47] Deary IJ, Weiss A, Batty GD (2010). Intelligence and personality as predictors of illness and death how researchers in differential psychology and chronic disease epidemiology are collaborating to understand and address health inequalities. Psychol Sci Public Interest.

[48] Kubzansky LD, Kawachi I, Sparrow D (1999). Socioeconomic status, hostility, and risk factor clustering in the normative aging study: any help from the concept of allostatic load?. Ann Behav Med.

[49] Graves KY, ACH N (2017). Childhood Socioeconomic Status and Stress in Late Adulthood: A Longitudinal Approach to Measuring Allostatic Load. Glob Pediatr Health.

[50] Barboza Solís C, Fantin R, Castagné R, Lang T, Delpierre C, Kelly-Irving M (2016). Mediating pathways between parental socio-economic position and allostatic load in mid-life: findings from the 1958 British birth cohort. Soc Sci Med.

[51] Zachau-Christiansen B, Ross EM. Babies: a study of human development during the first year: Wiley; 1975.

[52] Mortensen EL (1997). The Copenhagen perinatal cohort and the prenatal development project. Int J Risk Saf Med.

[53] Eysenck HJ, Eysenck SBG (1975). Manual of the Eysenck personality questionnaire.

[54] Wechsler D (1958). The Mearurement and appraisal of adult intelligence.

[55] Avlund K, Osler M, Mortensen EL, Christensen U, Bruunsgaard H, Holm-Pedersen P (2014). Copenhagen aging and midlife biobank (CAMB) an introduction. J Aging Health.

[56] Hansen ÅM, Lund R, Bruunsgaard H, Rod NH, Garde AH, Molbo D (2014). Social gradient in Allostatic load among Danish men and women in late midlife. J Aging Health.

[57] O’Donovan DD (1969). An historical review of the lie scale: with particular reference to the Maudsley personality inventory. Pap Psychol.

[58] Furnham A (1986). Response bias, social desirability and dissimulation. Personal Individ Differ.

[59] Christensen U, Krølner R, Nilsson CJ, Lyngbye PW, Hougaard CØ, Nygaard E (2014). Addressing social inequality in aging by the Danish occupational social class measurement. J Aging Health.

[60] Acock AC (2014). A gentle introduction to Stata.

[61] Bolger N, Zuckerman A (1995). A framework for studying personality in the stress process. J Pers Soc Psychol.

[62] Hawkley LC, Lavelle LA, Berntson GG, Cacioppo JT (2011). Mediators of the relationship between socioeconomic status and allostatic load in the Chicago health, aging, and social relations study (CHASRS). Psychophysiology.

[63] Friedman EM, Karlamangla AS, Gruenewald TL, Koretz B, Seeman TE (2015). Early life adversity and adult biological risk profiles. Psychosom Med.

[64] Singer B, Ryff CD (1999). Hierarchies of life histories and associated health risks. Ann N Y Acad Sci.

[65] Schaefer JD, Caspi A, Belsky DW, Harrington H, Houts R, Israel S (2016). Early-life intelligence predicts midlife biological age. J Gerontol B Psychol Sci Soc Sci..

[66] Hamdi NR, South SC, Krueger RF (2016). Does education lower allostatic load? A co-twin control study. Brain Behav Immun.

[67] Brännlund A, Hammarström A, Strandh M (2013). Education and health-behaviour among men and women in Sweden: a 27-year prospective cohort study. Scand J Public Health.

[68] Lodi-Smith J, Jackson J, Bogg T, Walton K, Wood D, Harms P (2010). Mechanisms of health: education and health-related behaviours partially mediate the relationship between conscientiousness and self-reported physical health. Psychol Health.

[69] Flouri E (2006). Parental interest in children’s education, children’s self-esteem and locus of control, and later educational attainment: twenty-six year follow-up of the 1970 British birth cohort. Br J Educ Psychol.

[70] Mckenzie SK, Carter KN, Blakely T, Ivory V (2011). Effects of childhood socioeconomic position on subjective health and health behaviours in adulthood: how much is mediated by adult socioeconomic position?. BMC Public Health.

[71] Seeman M, Stein Merkin S, Karlamangla A, Koretz B, Seeman T (2014). Social status and biological dysregulation: the “status syndrome” and allostatic load. Soc Sci Med.

[72] Lee-Baggley D, Preece M, DeLongis A (2005). Coping with interpersonal stress: role of big five traits. J Pers.

[73] von Stumm S, Macintyre S, Batty DG, Clark H, Deary IJ (2010). Intelligence, social class of origin, childhood behavior disturbance and education as predictors of status attainment in midlife in men: the Aberdeen children of the 1950s study. Intelligence.

[74] Gustafsson PE, Janlert U, Theorell T, Westerlund H, Hammarström A (2011). Socioeconomic status over the life course and allostatic load in adulthood: results from the northern Swedish cohort. J Epidemiol Community Health.

[75] Danese A, McEwen BS (2012). Adverse childhood experiences, allostasis, allostatic load, and age-related disease. Physiol Behav.

[76] Solís CB, Kelly-Irving M, Fantin R, Darnaudéry M, Torrisani J, Lang T (2015). Adverse childhood experiences and physiological wear-and-tear in midlife. Proc Natl Acad Sci U S A.

[77] Kuh D, Ben-Shlomo Y. Sicioeconomic pathways between childhood and adult health. In: Kuh D, Ben Shlomo Y, Ezra S, editors. A Life Course Approach to Chronic Disease Epidemiology: Oxford University Press; 2004. p. 371–96. http://www.oxfordscholarship.com/view/10.1093/acprof:oso/9780198578154.001.0001/acprof-9780198578154-chapter-16. Accessed 28 Jul 2016.

[78] Calvin CM, Batty GD, Lowe GDO, Deary IJ (2011). Childhood intelligence and midlife inflammatory and hemostatic biomarkers: the National Child Development Study (1958) cohort. Health Psychol Off J Div Health Psychol Am Psychol Assoc.

[79] Das A (2016). Psychosocial distress and inflammation: Which way does causality flow?. Soc Sci Med.

[80] Luciano M, Marioni RE, Gow AJ, Starr JM, Deary IJ (2009). Reverse causation in the association between C-reactive protein and fibrinogen levels and cognitive abilities in an aging sample. Psychosom Med.

[81] Juster R-P, McEwen BS, Lupien SJ (2010). Allostatic load biomarkers of chronic stress and impact on health and cognition. Neurosci Biobehav Rev.

