# Supplementary figures and images for: Parental socioeconomic position and midlife allostatic load: a study of potential mediators
Source: BMC Public Health. 2018 Aug 20;18:1029. doi: 10.1186/s12889-018-5956-x (PMC6102839; doi:10.1186/s12889-018-5956-x)

**Additional file 1. Figure S1.** Overview of data collection.

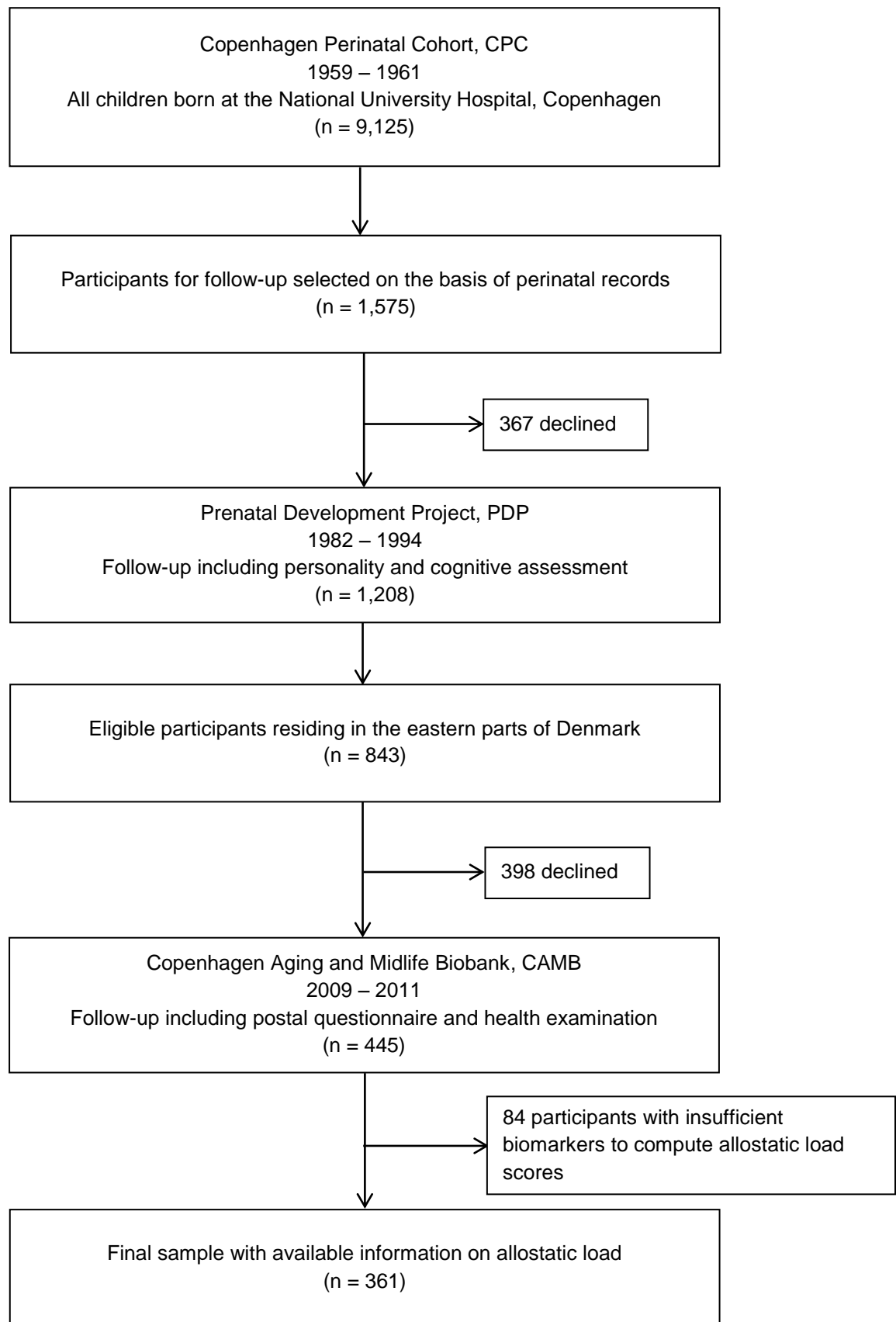

Supplement: Supplementary file 1 — Figure S1. Overview of the data collection. (PDF 73 kb) [file 12889_2018_5956_MOESM1_ESM.pdf]
